# Mechanistic insights and challenges in mitochondrial regulation of macrophage polarization and inflammatory responses

**DOI:** 10.3389/fphys.2026.1768672

**Published:** 2026-04-10

**Authors:** Xinran Liu, Renping Liu

**Affiliations:** Queen Mary College, Nanchang University, Nanchang, China

**Keywords:** inflammation, macrophage polarization, macrophages, metabolism, mitochondrial-regulated inflammation mechanisms

## Abstract

Inflammation has a dual nature; excessive or uncontrolled inflammation can trigger metabolic inflammatory diseases, in which immune cells, especially macrophages, play a crucial role. Mitochondria, as the core of cellular energy metabolism, are closely related to macrophage polarization and inflammation regulation. Mitochondrial dysfunction can trigger inflammatory responses through the activation of multiple signaling pathways, involving multiple signaling pathways, including Cyclic GMP-AMP Synthase – Stimulator of Interferon Genes 1, inflammasomes, and Retinoic acid-Inducible Gene I (RIG-I). Currently, the role of mitochondria in regulating inflammatory responses is increasingly prominent; however, current research still faces many challenges, such as a lack of mechanistic connections, unclear details of key molecules, insufficiently refined experimental strategies, and difficulties in clinical translation. Future research needs to leverage advanced technologies to delve deeper into the mechanisms, improve the bioavailability and tissue-specific delivery of mitochondrial-targeted drugs, establish personalized evaluation criteria, and promote interdisciplinary innovation to facilitate the transition of mitochondrial-targeted therapy from basic research to clinical application.

## 1 Introduction

Inflammation, as a defensive response of the body to external stimuli, possesses a dual nature. Under normal physiological conditions, the inflammatory response helps the body eliminate pathogens and repair damaged tissues, playing a positive role in maintaining homeostasis. However, when the inflammatory response is excessive or out of control, it can damage the body. Influenced by changes in modern lifestyles and environmental factors, the human body experiences metabolic disorders and abnormalities in various metabolic products. These changes further exacerbate chronic, low-level inflammatory responses in the body. Persistent inflammation can cause more severe damage to tissues and organs, leading to a series of metabolic inflammatory diseases, such as obesity, dementia, and atherosclerosis (Bektas et al., 2020; Tu et al., 2021). Taking dementia as an example, long-term, low-level inflammation in the brain is one of the key factors inducing and accelerating Alzheimer’s disease. Literature reports that the number of dementia patients worldwide increased to 43.8 million in 2016, a 117% increase compared to 20.2 million in 1990. It is estimated that by 2050, 100 million people will be suffering from dementia (GBD 2016 Dementia Collaborators, 2019). Dementia has become a major global public health problem, and its rapidly increasing number of patients places a heavy burden on families, society, and the economy. In 2019, the estimated global social burden caused by dementia was 1313.4 billion US dollars, and this number is growing at a rate of one case every 4 seconds (Zhong et al., 2025). Atherosclerotic cardiovascular disease (ASCVD) is equally concerning, primarily comprising ischemic heart disease (IHD) and cerebrovascular disease (mainly ischemic stroke). IHD and stroke are the leading and third leading causes of death globally, respectively. In 2013, these two diseases caused 247.9 deaths per 100,000 people, accounting for 84.5% of cardiovascular deaths and 28.2% of all-cause mortality (Barquera et al., 2015). In recent years, the burden of cardiovascular diseases has remained persistently high. Epidemiological data from Wang et al. indicate that about 19.42 million people died from cardiovascular diseases globally in 2021 (Wang et al., 2024). Among them, the number of deaths due to IHD increased significantly, reaching 8.99 million (Tan et al., 2025). These figures highlight the enormous threat atherosclerotic cardiovascular disease poses to global human health, and its high incidence and mortality rates make the prevention and treatment of this disease a crucial focus of global medical research. Immune cells, especially macrophages, play a crucial role in the initiation and subsequent development of metabolic inflammatory diseases. Macrophage polarization is regulated by metabolic stimuli, which in turn influence the evolution of metabolic dysfunction across multiple disease stages and in different tissues (Tian Q. et al., 2025). When faced with endogenous or exogenous stimuli, macrophages differentiate into various phenotypes. Currently, the widely accepted classification divides macrophages into classical/inflammatory (M1) activated macrophages and alternating/anti-inflammatory (M2) activated macrophages (Zhou et al., 2018). However, under numerous physiological and pathological conditions, macrophage polarization is not a simple binary state but a continuous process. This process reflects the high plasticity and heterogeneity of these effector cells, which behave more like a collection of cells in different polarization states, making it difficult to summarize using the overly simplistic M1/M2 classification.

Mitochondria play a pivotal role in cellular energy metabolism. The process of mitochondrial energy conversion commences with the tricarboxylic acid cycle (TCA) occurring within the mitochondrial matrix. During this cycle, energy is liberated through the oxidation of acetyl-CoA, leading to the reduction of nicotinamide adenine dinucleotide (NAD+) to its reduced form, Nicotinamide Adenine Dinucleotide (NADH). Subsequently, NADH enters the oxidative phosphorylation (OXPHOS) pathway, where electrons are shuttled through the electron transport chain embedded in the mitochondrial inner membrane. This electron transfer drives the pumping of protons, thereby establishing a proton gradient. The resultant proton motive force facilitates the rotation of the F0-F1-ATP synthase complex, ultimately catalyzing the synthesis of Adenosine Triphosphate (ATP) (Mourokh and Friedman, 2024). ATP is an indispensable energy molecule for cellular activity, providing power for various physiological functions. A shortage of intracellular ATP can damage organs and tissues with extremely high energy demands, such as the heart, muscles, and brain, potentially leading to a series of serious diseases and threatening overall health (Johnson et al., 2019). Current research on inflammation largely focuses on exploring and describing the characteristics of cellular and humoral immunity (Yu et al., 2024). However, the impact of inflammation and the anti-inflammatory environment on macrophage mitochondrial function remains underdeveloped. Therefore, we discuss the role of macrophage mitochondrial function in inflammation, hoping to provide insights for the prevention and treatment of inflammatory diseases.

## 2 Overview of macrophages

### 2.1 Macrophage polarization

Macrophages are infiltrative immune cells originating from the bone marrow mononuclear cell system (Dash et al., 2024; Richards et al., 2013). When the body is stimulated by inflammation, mononuclear cells migrate to inflamed tissues via the bloodstream and differentiate into macrophages under the influence of local growth factors, pro-inflammatory cytokines, and microbial products (Shi and Pamer, 2011). Macrophages exhibit high plasticity and dynamism, and their phenotype and function can change according to specific biological functions and pathophysiological processes (**Table 1**). They participate in the occurrence, persistence, and resolution of inflammation by secreting bioactive molecules; this process is known as macrophage polarization, which is mainly divided into the classically activated M1 phenotype and the alternately activated M2 phenotype (Sheu and Hoffmann, 2022; Wang et al., 2021). An imbalance in the M1/M2 polarization state, such as abnormal activation of M1 and stagnation of M1-M2 conversion, can lead to a chronic inflammatory state and trigger various diseases (Cutolo et al., 2022). The traditional M1/M2 dichotomy has long served as a cornerstone framework for elucidating macrophage function; nevertheless, this established paradigm is now facing mounting challenges from the advent of single-cell and spatial transcriptomics technologies (Kumar Jha et al., 2024). These cutting-edge methodologies have unveiled that macrophages do not conform to distinct bipolar states but rather exist along a continuous, fluid phenotypic spectrum. Variations in their developmental origins and microenvironmental signals propel macrophages to assume diverse activation states that transcend the simplistic M1/M2 classification (Palma, 2025). While all subsets of tissue-resident macrophages trace their origins back to a shared myeloid progenitor, they migrate to and colonize specific tissues during embryonic development, giving rise to specialized populations finely attuned to their respective microenvironments—such as Kupffer cells in the liver, microglial cells in the central nervous system, and osteoclasts in bone tissue. In pathological settings, including inflammatory diseases and atherosclerosis, macrophages respond to a complex interplay of lipid, cytokine, and metabolic cues, differentiating into phenotypes that are uniquely tailored to the disease context. Within atherosclerotic plaques, in particular, specialized macrophage subpopulations emerge that extend beyond the classical M1 and M2 classifications: Mox macrophages, distinguished by their antioxidant properties, and Mhem macrophages, which exhibit anti-atherosclerotic functions and are adept at erythrocyte phagocytosis (Chistiakov et al., 2015; Li et al., 2022). These macrophage subsets play a pivotal role in modulating plaque stability and the formation of necrotic cores—processes that the conventional M1/M2 dichotomy fails to adequately encapsulate. This limitation highlights the imperative for a more nuanced and comprehensive understanding of macrophage heterogeneity in both healthy and diseased states.

**Table 1:** Macrophage phenotypes and functions.

Phenotype	Function	Stimulating factor	Secretory factor	References
M1	It promotes inflammatory responses, fights pathogens, and has antitumor effects.	Lipopolysaccharide (LPS), Interleukin-12 (IL-12), IL-23, Tumor Necrosis Factor-alpha (TNF-α)	IL-1β, IL-6, TNF-α, IL-12	(Shapouri-Moghaddam et al., 2018; Yunna et al., 2020; Zhou et al., 2024)
M2​				
M2a	It has functions including immune regulation, tumor promotion, involvement in allergic reactions, and parasitic infections.	IL-4, IL-13	IL-10, Tumor Necrosis Factor-β (TGF-β), Insulin-like Growth Factor (IGF)	(Colin et al., 2014; Funes et al., 2018)
M2b	It has dual pro-inflammatory and anti-inflammatory effects, suppresses immune responses, and promotes tissue repair and tumor progression.	Toll-like Receptor (TLR)	IL-1, IL-6, C-C motif chemokine ligand 1 (CCL1), CCL20	(Guan et al., 2025)
M2c	Immunosuppression, tissue remodeling.	IL-10, TGF-β	TGF-β, IL-10, CCL18	(Funes et al., 2018)
M2d	Promotes tumor growth and angiogenesis.	Adenosine A2A receptor (A2AR)	Vascular Endothelial Growth Factor (VEGF), IL-10	(Ferrante and Leibovich, 2012; Grinberg et al., 2009)
M4	Pro-inflammatory response, low phagocytosis	C-X-C motif chemokine ligand 4 (CXCL4)	IL-6, TNF-α	(Erbel et al., 2015)
Mox	Low chemotaxis and low phagocytosis	Oxidized Phospholipids (OxPLs)	IL-1β, IL-10	(Colin et al., 2014; Kadl et al., 2010)
​M(Hb) (hemoglobin-stimulated macrophages)	Low chemotaxis and low phagocytosis	Hemoglobin	ATP-binding cassette transporter A1 (ABCA1), ATP-binding cassette transporter G1 (ABCG1), Liver X receptor alpha (LXRα)	(Colin et al., 2014; Finn et al., 2012)

In the early phase, unpolarized macrophages (M0 macrophages) undergo polarization into M1 macrophages under the influence of a pro-inflammatory microenvironment. This transformation is triggered by stimuli including Type 1 T helper cell-associated cytokines (Th1 cytokines), such as Interferon-gamma and TNF-α, as well as bacterial products like lipopolysaccharide (LPS) and viral infections (Bosco, 2019). M1 macrophages have strong antigen-presenting capabilities, eliminating pathogens and protecting the host by releasing high levels of pro-inflammatory cytokines (TNF-α, IL-1β, IL-6, Cyclooxygenase-2) and low levels of IL-10. They can also activate the Nicotinamide Adenine Dinucleotide Phosphate (NADPH) oxidase system to produce reactive oxygen species (ROS) and participate in redox reactions (Kerneur et al., 2022; Lampiasi, 2022; Saeedifar et al., 2021). However, excessive release of pro-inflammatory factors and ROS may mediate tissue damage and affect wound healing and tissue regeneration. To avoid a prolonged state of chronic inflammation, M0 and M1 macrophages polarize into M2 macrophages with anti-inflammatory activity under the stimulation of Type 2 T helper cell-related cytokines (Th2 cytokines) and immune complexes (Deng et al., 2023). Based on different activating stimuli, M2 macrophages can be further divided into four subsets: M2a, M2b, M2c, and M2d (Bucher et al., 2022). M2a macrophages are activated by interleukin-4 (IL-4) and interleukin-13 (IL-13); M2b macrophages are induced by IgG immune complexes in conjunction with Toll-like receptor agonists; M2c macrophages are triggered by glucocorticoids or Macrophage Colony-Stimulating Factor (M-CSF) in an IL-10-dependent manner; and M2d macrophages are induced by the combined action of interleukin-6 (IL-6), Toll-like receptor stimulation, and A2 adenosine receptors (Xu et al., 2025). Following polarization, M2 macrophages secrete anti-inflammatory cytokines, including interleukin-10 (IL-10) and transforming growth factor-beta (TGF-β). Their primary roles encompass facilitating tissue repair, accelerating wound healing, fostering angiogenesis, and contributing to fibrosis processes (Horibe et al., 2025).

### 2.2 The correlation between the dynamic equilibrium of M1/M2 polarization and the inflammatory response

Inflammation, as a sophisticated defense mechanism for maintaining homeostasis, can be triggered by damaged cells, pathogens, and stimuli. However, dysregulation can lead to adverse consequences such as chronic inflammation and cardiovascular disease (Bellanti et al., 2025). Macrophage polarization is crucial in the inflammatory response and is regulated by cytokines. In atherosclerosis models, the polarization state of M1/M2 macrophages exhibits dynamic changes during disease progression and regression. M1 dominates during the progression phase, while M2 dominates during plaque stabilization. The transition from M2 to M1 exacerbates atherosclerosis (Peled and Fisher, 2014; Shrivastava et al., 2019). Li et al. found that mesenchymal stem cell-derived exosomes can increase the infiltration and polarization of M2 macrophages in Apoe^-^/^-^ mice, thereby reducing plaque area (Li et al., 2019). In rheumatoid arthritis research, M1/M2 polarization imbalance leads to acute or chronic rheumatoid arthritis (Cutolo et al., 2022). Kim et al., using a mouse model, employed M2 macrophage-derived vesicles rich in factors related to M2 generation and macrophage reprogramming to drive synovial macrophage polarization from M1 to M2, effectively alleviating joint damage and inflammatory responses in mice (Kim et al., 2022). In lupus nephritis research, increasing the frequency of M2 macrophages and enhancing anti-inflammatory properties can alleviate lupus nephritis. Liang et al., using a Pristan-induced mouse model, found that total peony glucoside (TGP) can efficiently increase the frequency of M2 macrophages through the IL-4-mediated Signal Transducer and Activator of Transcription 6 (STAT6) signaling pathway and improve kidney damage in mice through its anti-inflammatory effects (Liang et al., 2021). In studies of inflammatory bowel disease (IBD), Daskalaki et al. pointed out that neorogioltriol can inhibit M1 and promote M2 responses to limit the progression of IBD (Daskalaki et al., 2019). In summary, maintaining the balance of M1 and M2 macrophage phenotypes is an effective and important strategy for treating inflammatory diseases.

## 3 Overview of mitochondria

### 3.1 Mitochondrial structure and function

**Figure 1:**
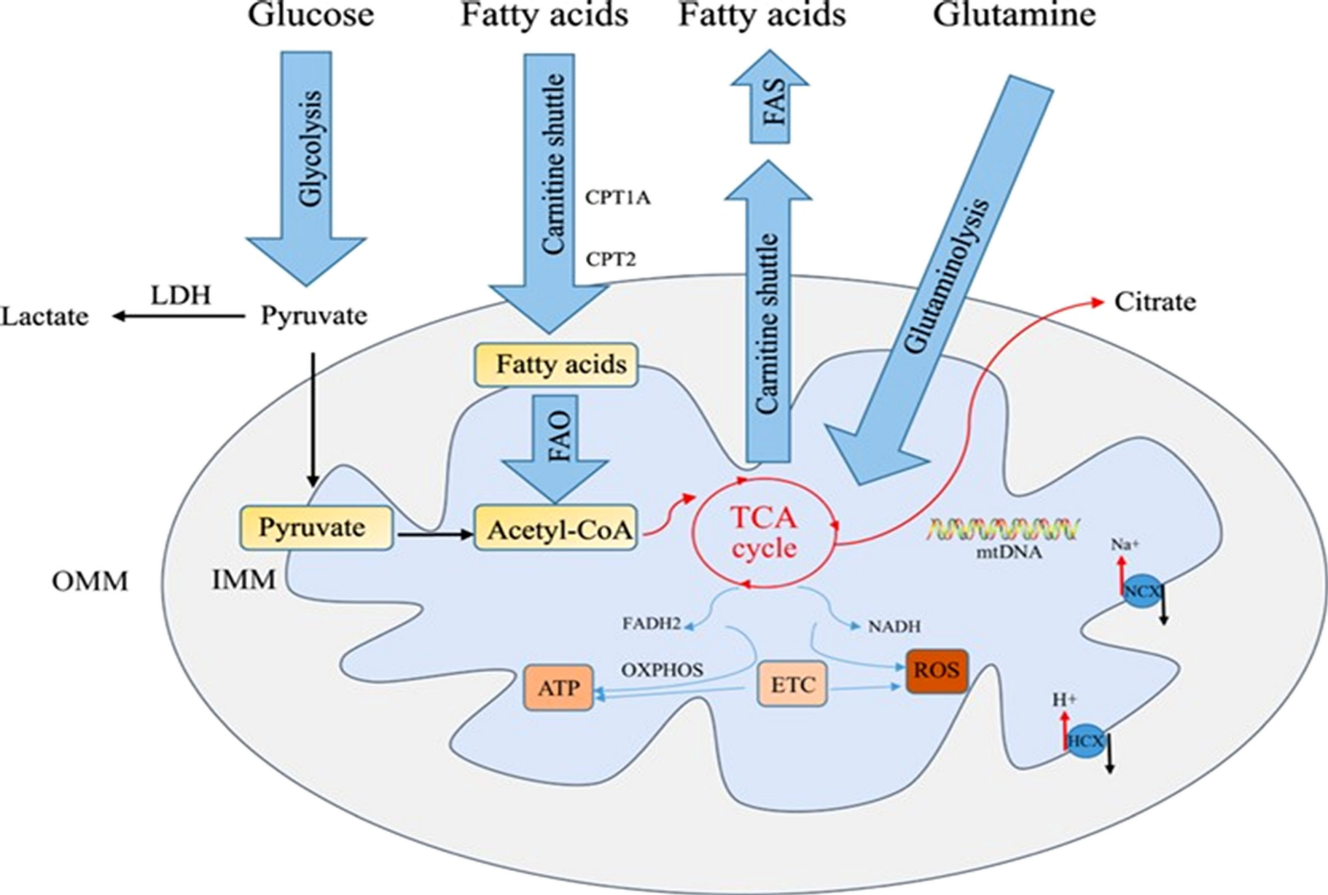
Mitochondrial metabolic pathway (Qing et al., 2020).

Mitochondria serve as indispensable organelles within cells, characterized by a complex structural composition that encompasses the outer mitochondrial membrane, inner mitochondrial membrane, intermembrane space, mitochondrial matrix, distinct mitochondrial DNA (mtDNA), and mitochondrial-associated membranes (MAMs). Collectively, these components form the structural foundation upon which mitochondrial function is built (Liu et al., 2025). They are indispensable for maintaining normal cellular physiological functions. Besides synthesizing ATP, the cell’s energy “currency,” mitochondria are deeply involved in various physiological processes, such as intracellular calcium ion (Ca²^+^) signaling, stress and metabolic signal transduction, apoptosis, and participation in various metabolic reactions (Rossi et al., 2019) (**Figure 1**). Both the outer mitochondrial membrane (OMM) and inner mitochondrial membrane (IMM) are composed of a phospholipid bilayer. The outer membrane separates the internal mitochondrial contents from the cytoplasm; small molecules and ions can freely enter and exit the mitochondria through the outer membrane pores, while large molecules rely on membrane transporters for transmembrane transport. The protein composition of the inner mitochondrial membrane is exceedingly intricate, encompassing not only the complexes of the electron transport chain (ETC) and inner membrane translocases (TIMs) but also being richly endowed with ATP synthase, proteins involved in mitochondrial dynamics, ion transport and metabolic carrier proteins, proteins related to mitochondrial fusion and fission, as well as protein complexes implicated in oxidative stress response and apoptotic regulation. These diverse components collectively uphold mitochondrial homeostasis and orchestrate its functional regulation (Ahmad et al., 2018; Kizmaz et al., 2024; Kunji et al., 2025; Padh et al., 2021; Plouzennec et al., 2025). The TIM (Translocase of the Inner Mitochondrial Membrane) complex plays a pivotal role in facilitating the translocation of cytoplasmically synthesized proteins into the mitochondrial matrix, a process that hinges on both the mitochondrial inner membrane potential and ATP hydrolysis. Concurrently, the electron transport chain (ETC) generates an electrochemical gradient by actively pumping protons across the inner mitochondrial membrane, thereby providing the driving force for ATP synthase to catalyze ATP synthesis. Together, these mechanisms ensure the proper maintenance of mitochondrial energy metabolism (Ahmad et al., 2018; Kizmaz et al., 2024). The inner mitochondrial membrane folds inward to form cristae, a structural adaptation that not only significantly expands its surface area, thereby offering abundant space for ATP synthesis, but also establishes specialized microdomains that harbor both active and inactive ATP synthase complexes (Domínguez-Zorita et al., 2022).The mitochondrial matrix, located within the inner membrane, contains various enzymes and proteins involved in metabolic processes such as mitochondrial oxidative phosphorylation and fatty acid oxidation (FAO) (Wang et al., 2019). Furthermore, mitochondria participate in the synthesis of cell membrane components, crucial for maintaining cellular stability. mtDNA plays a decisive role in mitochondrial function and self-replication, encoding key proteins required by mitochondria to ensure accurate replication and transmission of genetic information. Many diseases, such as inflammatory diseases and age-related symptoms, are closely related to mtDNA damage or mutations (Sanchez-Contreras and Kennedy, 2022). Therefore, in-depth research into the encoding mechanisms and functions of mtDNA can help unravel the mysteries of life and provide new insights for disease prevention and treatment.

This figure depicts the core metabolic machinery within mitochondria. The double-membrane structure is annotated: outer membrane with porins, inner membrane forming cristae to maximize surface area, intermembrane space, and matrix. The tricarboxylic acid cycle (TCA cycle) occurs in the matrix, where acetyl-CoA is oxidized via citrate synthase, isocitrate dehydrogenase, and α-ketoglutarate dehydrogenase to generate NADH and FADH_2_. The oxidative phosphorylation (OXPHOS) system on the inner membrane comprises Complex I-IV of the electron transport chain (ETC), which transfers electrons from NADH/FADH_2_ to O_2_, creating a proton gradient that drives ATP synthase (Complex V) to produce ATP. Key nodes are highlighted: cytochrome c shuttling between Complex III/IV, and the rotational catalysis of ATP synthase’s F_1_ subunit. This pathway exemplifies mitochondria as cellular powerhouses, regulated by mtDNA mutations, reactive oxygen species (ROS), and nutrient availability.

### 3.2 Mitochondrial homeostasis and its regulatory mechanisms

The maintenance and dynamic remodeling of mitochondrial homeostasis are crucial for normal cellular physiological functions. Their state can be measured by several key indicators: (1) In terms of quantity and size, the number of mitochondria varies depending on cell type and energy demand. Increased energy demand leads to a higher abundance of mitochondria (Tandler and Erlandson, 1983). Energy demand fluctuates, and the number and volume are adjusted through fusion/division and biogenesis (Puleston, 2015). In terms of morphology and structure, normal mitochondria exhibit a typical double-membrane structure, with a smooth outer membrane and an inner membrane folded into cristae, appearing elliptical or rod-shaped with a dynamic network structure. During disease or aging, they fragment, the cristae become irregular, and their number decreases. Increased energy demand or cell replication and division lead to fusion, forming longer structures and denser cristae to enhance energy production efficiency (Awad and Abdul Karim, 2025; Babbitt et al., 2015; Lyra-Leite et al., 2021; Qin and Xi, 2022; Vasileiou et al., 2019; Zaninello et al., 2024); A decrease in membrane potential (MMP) often indicates uncoupling of oxidative phosphorylation or opening of the membrane permeability transition pore, which is directly related to the initiation of apoptosis (Wang et al., 2023); Superoxide anions produced within mitochondria are transformed into Hydrogen Peroxide (H_2_O_2_) by Superoxide Dismutase 2 (SOD2). Subsequently, the H_2_O_2_ is primarily detoxified by peroxidases and, to a lesser degree, by glutathione peroxidases (García‐Ruiz and Fernández‐Checa, 2018; Lee et al., 2026; Lindsay and Rhodes, 2025). Low levels of ROS regulate cell proliferation and differentiation, while high concentrations may activate inflammasomes or damage DNA (Huang et al., 2020; Mo et al., 2019; Zhang et al., 2024); The copy number of mtDNA is dynamically adjusted with energy demand, and its mutation accumulation is associated with a variety of diseases.

**Figure 2:**
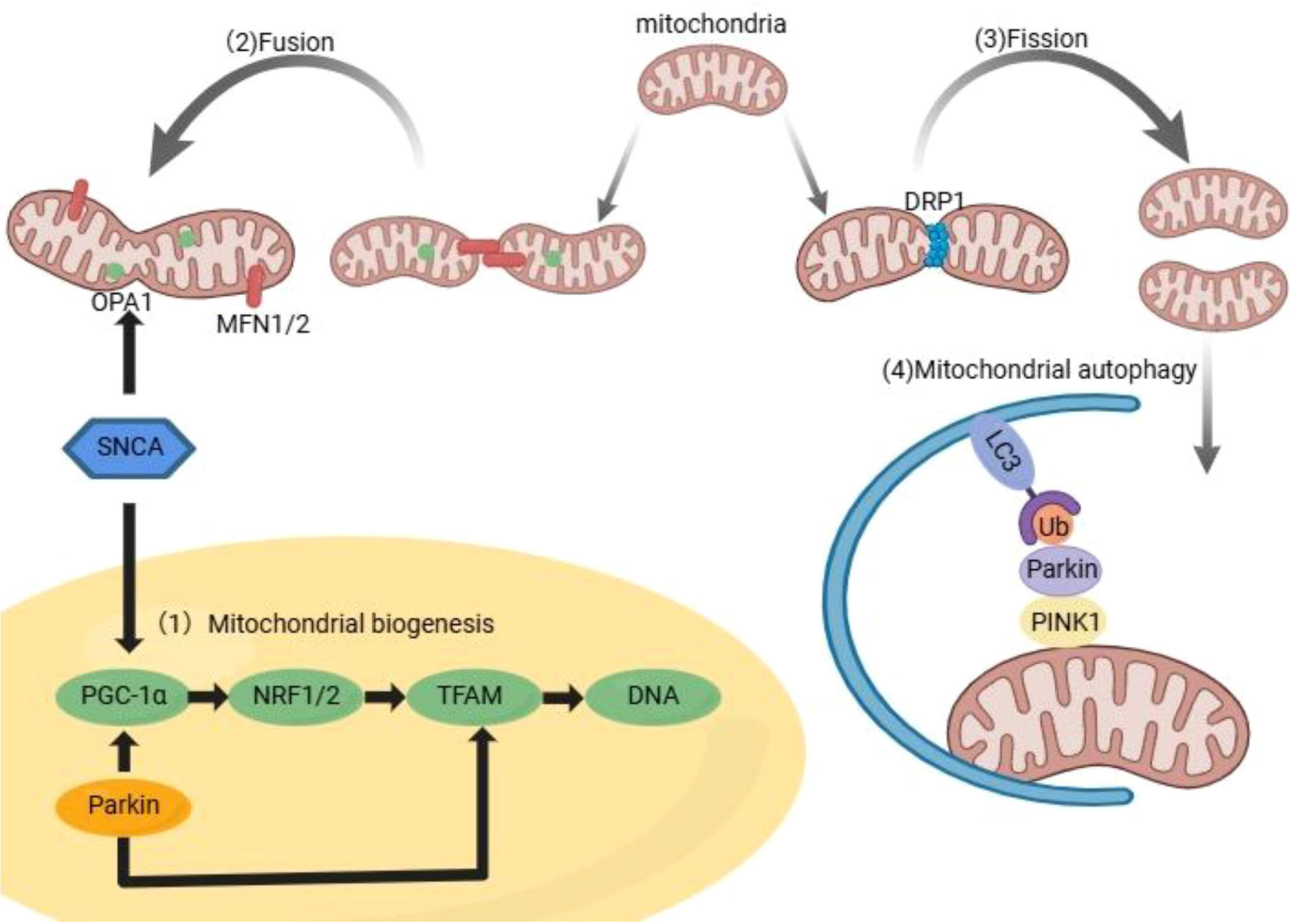
Overview of mitochondrial quality control pathways and core regulators.

Recent in-depth research has revealed that mitochondria maintain their homeostasis through a multidimensional regulatory network comprised of biogenesis, fission and fusion, and autophagy (**Figure 2**). Mitophagy selectively clears dysfunctional and damaged mitochondria via the PTEN-induced putative kinase 1- Parkin RBR E3 ubiquitin protein ligase (PINK1-Parkin) pathway, maintaining a balance between quantity and quality. Under physiological conditions, autophagy clears impaired mitochondria to maintain energy metabolism homeostasis; under pathological conditions such as oxidative stress, autophagy flux is abnormally upregulated, triggering a programmed cell death cascade. Over-activated autophagy-apoptosis molecular switches are significantly associated with neurodegenerative diseases and chemotherapy resistance (Yi et al., 2020). Clinically, patients with polycystic ovary syndrome (PCOS) have increased expression of mitochondrial autophagy-related markers in granulosa cells, and melatonin can reduce their expression (Deng et al., 2024; Yi et al., 2020). Mitochondrial biogenesis is a complex and precise dynamic equilibrium process encompassing mtDNA replication, cristae remodeling, and quantitative regulation. It is synergistically regulated by multiple signaling pathways, with Peroxisome proliferator-activated receptor gamma coactivator 1-alpha (PGC-1α) being a core regulatory factor. PGC-1α promotes mtDNA replication and mitochondrial protein expression by activating Mitochondrial transcription factor A (TFAM), synergistically regulating the expression of respiratory chain complex subunits. During cellular energy stress, upregulation of PGC-1α transcription drives mitochondrial remodeling, enhancing metabolic function to meet energy demands (Fontecha-Barriuso et al., 2020). Mitochondrial fission and fusion achieve dynamic remodeling of organelle morphological networks through kinetic equilibrium, precisely regulating mitochondrial population heterogeneity (Adebayo et al., 2021). Mitochondrial fission is predominantly governed by the cytosolic Guanosine triphosphatase (GTPase) Dynamin-related protein 1 (DRP1). This protein is recruited to the outer mitochondrial membrane (OMM) via interactions with its receptors, such as Mitochondrial fission 1 protein (FIS1) and Mitochondrial fission factor (MFF) (Losón et al., 2013). Overactivation of DRP1 induces abnormal mitochondrial fragmentation, which facilitates the release of mitochondrial DNA (mtDNA) fragments and subsequently triggers inflammatory responses (Jia et al., 2025; Sui et al., 2022; Zanfardino et al., 2025). Elevated expression of DRP1 results in excessive mitochondrial fission and impedes mitochondrial fusion. This DRP1-mediated aberrant fission leads to the accumulation of fragmented mitochondria within neurons, thereby heightening the susceptibility to Alzheimer’s disease (Gao et al., 2022). In myocardial ischemia-reperfusion injury, downregulation of Mitofusin 2 (MFN2) expression exacerbates mitochondrial fragmentation, promotes ROS bursts, and induces cell death (Zhu et al., 2021). However, a growing body of evidence indicates that DRP1 may exhibit context-specific anti-inflammatory effects under ischemic conditions. Recent research has demonstrated that, in response to ischemic stress, DRP1-driven mitochondrial fission in macrophages can curb excessive inflammation by facilitating mitophagy and preventing the overproduction of mitochondrial reactive oxygen species (ROS), thereby preserving immune homeostasis (Yadav et al., 2025). This dual - faceted functionality underscores the imperative for a nuanced and in - depth interpretation of DRP1’s role in diverse pathological scenarios.

This schematic illustrates four key processes of mitochondrial homeostasis: (1) Mitochondrial biogenesis, regulated by Parkin via the PGC−1α–NRF1/2–TFAM axis to drive mtDNA synthesis; (2) Fusion, mediated by OPA1 and MFN1/2, occurring under high energy demand; (3) Fission, driven by DRP1 to segregate damaged segments; and (4) PINK1–Parkin-mediated mitophagy, which selectively clears dysfunctional mitochondria via ubiquitination and LC3-dependent autophagy, balancing mitochondrial number and quality to maintain cellular health. Abbreviations: NRF1/2, nuclear respiratory factors 1 and 2; LC3, microtubule−associated proteins 1A/1B light chain 3; SNCA, α−synuclein; OPA1, optic atrophy 1.

## 4 Mitochondrial homeostasis and macrophage polarization

**Figure 3:**
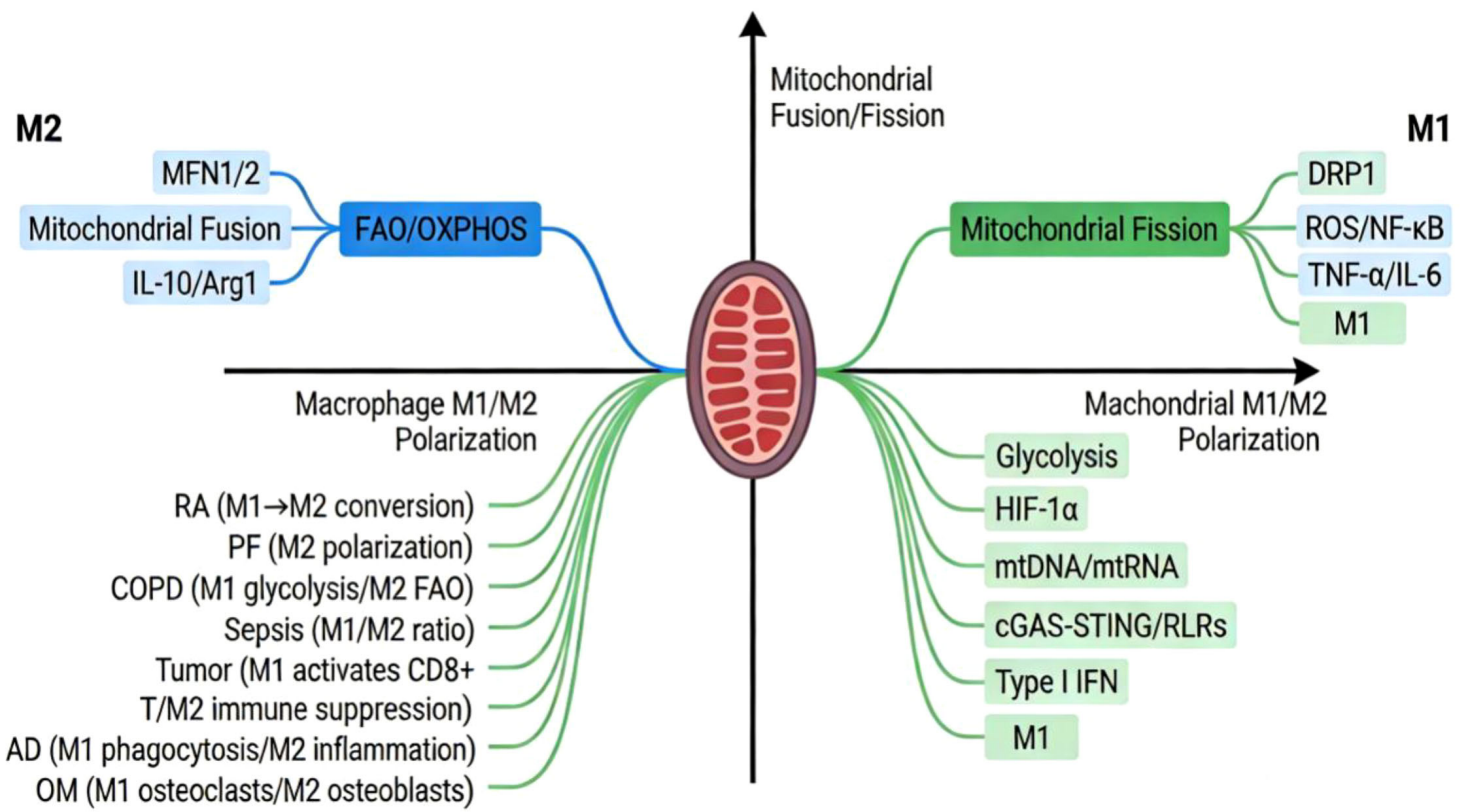
A cross-disease molecular mechanism map of mitochondrial dynamic regulation of macrophage polarization.

As a key intersection of cellular energy metabolism and immune regulation, mitochondria play a profound role in macrophage polarization regulation through dynamic remodeling of homeostasis, primarily focusing on three dimensions: topological conformation, population size, and metabolic mode switching (**Figure 3**). In terms of topological conformation, increased DRP1 phosphorylation leads to mitochondrial fragmentation, triggering ROS bursts, activating the Nuclear factor kappa-light-chain-enhancer of activated B cells (NF-κB) signaling pathway, upregulating the expression of pro-inflammatory factors such as TNF-α and IL-6, and driving macrophages towards M1 polarization (Deva Nathan, 2023). Conversely, MFN2 deficiency leads to mitochondrial fragmentation. This is because macrophage M2 polarization depends on the extended network structure formed by mitochondrial fusion, which supports FAO. MFN2 deficiency disrupts this structure, thus inhibiting the expression of M2 macrophage markers arginase 1 (Arg1) and interleukin-10 (IL-10). Enhanced mitophagy facilitates the polarization of macrophages toward the M2 phenotype, which is intricately linked to anti-inflammatory responses and tissue regeneration, ultimately contributing to the amelioration of inflammation (Vafadar et al., 2024); while Parkin deficiency inhibits autophagy, leading to the accumulation of damaged mitochondria. Damaged mitochondria release mtDNA, which drives macrophages toward M1 polarization via the Cyclic GMP-AMP Synthase–Stimulator of Interferon Genes (cGAS-STING) signaling pathway, thereby exacerbating the inflammatory response (He et al., 2024; Zhong et al., 2022). Mitochondrial biogenesis governs the mtDNA copy number, a factor of paramount importance for M2 macrophage polarization, via the PGC-1α/Nuclear Respiratory Factor 1 (NRF1) signaling axis (Abu Shelbayeh et al., 2023; Játiva et al., 2022). In terms of metabolic patterns, M1 polarization depends on glycolysis, which inhibits OXPHOS, leading to succinate accumulation, stabilizing Hypoxia-inducible factor 1-alpha (HIF-1α), and thus promoting IL-1β transcription (Tannahill et al., 2013; Viola et al., 2019). M2 macrophage polarization is critically dependent on fatty acid oxidation (FAO), a metabolic pathway that generates acetyl-CoA to fuel the tricarboxylic acid (TCA) cycle and sustain oxidative metabolism (Geeraerts et al., 2017; Liu et al., 2026). This process is further reinforced by AMP-activated protein kinase (AMPK) activation, which enhances FAO and mitochondrial biogenesis through the induction of peroxisome proliferator-activated receptor gamma coactivator 1-alpha (PGC-1α). Consequently, this metabolic reprogramming amplifies electron transport chain (ETC) activity, thereby solidifying the oxidative metabolic signature essential for M2 macrophage polarization (Cui et al., 2023; Maruta et al., 2022; Ramos-Jiménez et al., 2025; Zhou et al., 2023). This metabolic plasticity makes mitochondria a core regulator of macrophage polarization (Li et al., 2025a).

**Figure 4:**
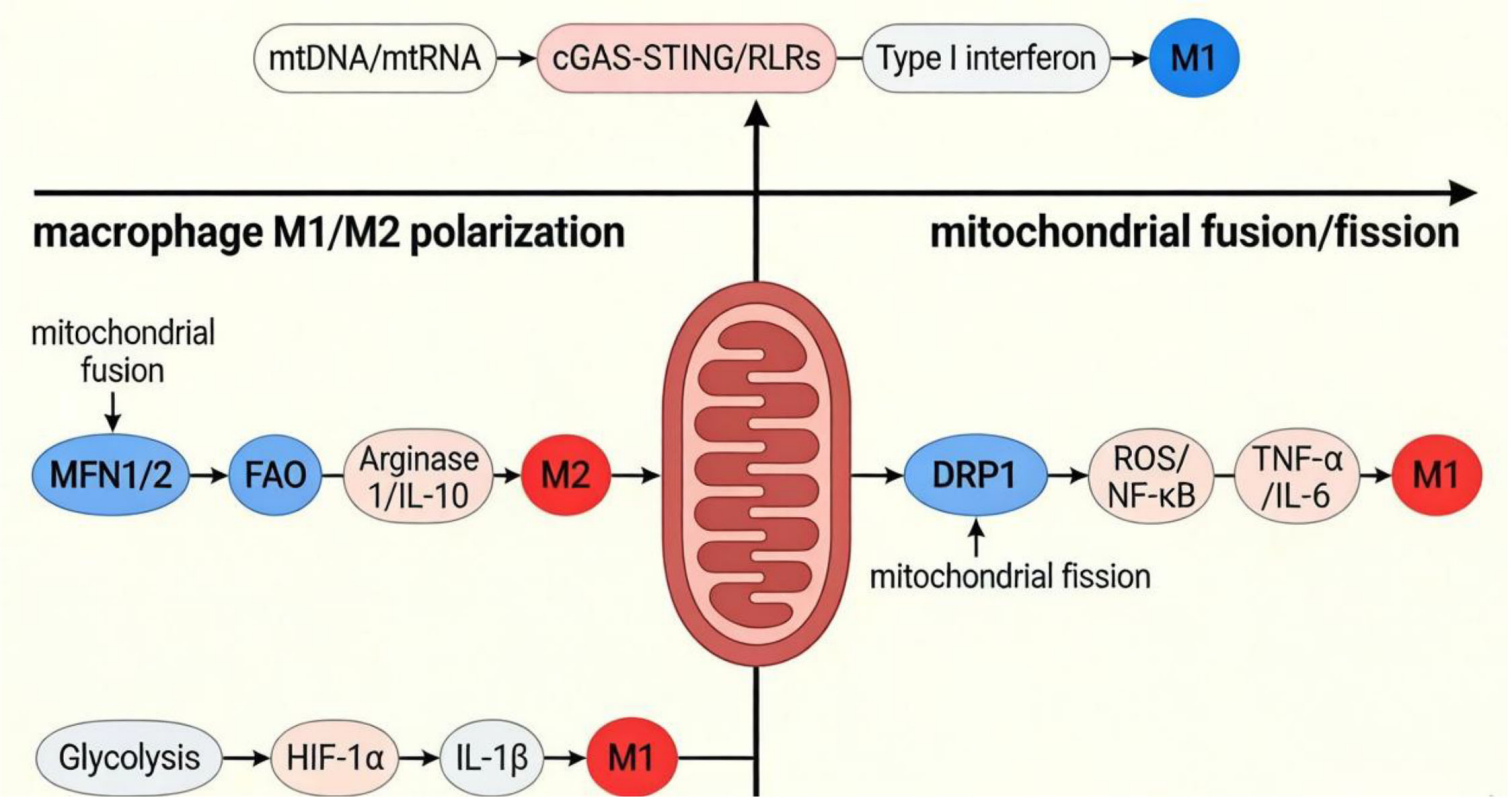
Schematic diagram of the molecular mechanism by which mitochondria dynamically regulate macrophage M1/M2 polarization.

Compared to single signaling pathways, mitochondria possess a multi-level integration advantage in regulating macrophage polarization. Located at a critical intersection of metabolism and immunity, they can coordinate energy status, redox balance, and inflammatory signals, simultaneously sense cellular microenvironment nutrition and pathogen threats and respond rapidly. Intervening in their related mechanisms provides a feasible approach for early regulation of macrophage polarization (Ahmed et al., 2019; Li et al., 2025a; Marques et al., 2024; Ryu et al., 2024). Targeted interventions focused on mitochondria offer precise control over the function of specific subcellular compartments. For instance, the mitochondrial antioxidant Mitoquinol (MitoQ) is capable of selectively scavenging mitochondrial reactive oxygen species (ROS) without disrupting the redox balance in the cytoplasm. In an atherosclerosis model, MitoQ demonstrates the ability to specifically suppress macrophage polarization toward the pro-inflammatory M1 phenotype, thereby reducing inflammatory cell infiltration within atherosclerotic plaques. Moreover, it exhibits superior organ-protective effects compared to conventional anti-inflammatory medications (Li et al., 2025a; Mercer et al., 2012). Mitochondrial dynamics proteins have multiple effects. DRP1 not only regulates mitochondrial division but can also bind to Mitochondrial antiviral signaling (MAVS) to enhance the RIG-I signaling pathway and form an antiviral response channel (Chen et al., 2020). Beyond its role in mediating mitochondrial fusion, MFN2 also regulates calcium ion flux through Endoplasmic Reticulum-Mitochondria Associated Membranes (ER-MAMs), thereby influencing Nuclear Factor of Activated T cells (NFAT) signaling pathways. This makes mitochondrial-based targeted regulatory strategies more efficient in terms of spatiotemporal coordination (Das et al., 2022; Zhao et al., 2012) (**Figure 4**).

## 5 Molecular mechanisms of mitochondrial regulation of inflammation

In recent years, mitochondria have been recognized as pivotal regulators of inflammatory responses, primarily due to their release of diverse damage-associated molecular patterns (DAMPs) and their role as physical scaffolds for activating specific pattern recognition receptors (PRRs) (Mehta et al., 2017). Mitochondrial dysfunction is a crucial factor triggering inflammatory responses, involving multiple signal transduction cascades. Among these, the cGAS-STING1 signaling pathway activated by mtDNA and the inflammasome signaling pathway induced by mtDNA and ROS have attracted considerable attention (**Figure 2**). These pathways precisely regulate the occurrence and development of inflammatory responses in response to internal and external stimuli, providing new perspectives for understanding the pathogenesis of inflammation-related diseases.

### 5.1 cGAS-STING1 pathway

In cGAS-STING1 signaling, cGAS is highly sensitive to double-stranded DNA (dsDNA) in the cytoplasm and can catalyze the formation of circular GMP-AMP (cGAMP), thereby initiating an inflammatory response (Decout et al., 2021). Mitochondrial dysfunction-induced changes in mitochondrial outer membrane permeability (MOMP) can allow mtDNA to enter the cytoplasm and activate cGAS, but this process is inhibited by apoptotic caspases (De Gaetano et al., 2021; Jenner and Garcia-Saez, 2024; Rongvaux et al., 2014; West, 2015; White et al., 2014). Different forms of dsDNA have varying abilities to activate cGAS, and infection by various intracellular pathogens can interfere with this process. Furthermore, there exists a BCL2-associated X protein (BAX) and BCL2 homologous antagonist/killer 1 (BAK1)-independent mtDNA release mechanism (Eaglesham and Kranzusch, 2020; Kim et al., 2019; Liu and Xu, 2025; Luecke et al., 2017; Pérez et al., 2024). Flores-Romero et al. observed that in specific contexts of Shigella infection of human cells, the proteolytically activated BH3 domain death agonist (BID) functions as a mitochondrial porogen, rather than acting as an activator of BAX and BAK1 as traditionally understood (Flores‐Romero et al., 2022). Furthermore, Kim’s experiments revealed that mild mitochondrial stress, which does not induce mitochondrial outer membrane permeability (MOMP) or regulated cell death (RCD), achieves mtDNA release through a Voltage-dependent anion channel (VDAC)-dependent mechanism (Kim et al., 2019). Concurrently, mtDNA enters the cytoplasm through the mitochondrial permeability transition pore (mPTP) located on the inner mitochondrial membrane (VanPortfliet et al., 2024). These findings illustrate the complexity of mtDNA activation of the cGAS-STING1 pathway. Despite unknowns, numerous studies have shown that mtDNA binding to cGAS and STING1 can effectively drive inflammatory responses when caspase inhibition is limited (Danthi, 2016).

### 5.2 Inflammasomes

Inflammasomes, as supramolecular platforms for activating specific caspases, play a crucial role in inflammatory responses. Oxidized mtDNA released by mitochondrial dysfunction is a potent activator of the NLRP3 inflammasome. ROS form a feedforward loop that promotes mtDNA release, and optimal signal transduction of the NLRP3 inflammasome involves multiple molecular interactions (Cabral et al., 2023; Jaara and Torres, 2024; Saxena et al., 2010; Wu et al., 2024; Zhou et al., 2010). Furthermore, mtDNA can also activate inflammasomes containing melanoma deficiency factor 2 (AIM2) (Boengiu et al., 2025). Different inflammasomes differ in recognizing DNA oxidation states, but oxidation is crucial for mtDNA-induced inflammasome signal transduction. Crane et al. first demonstrated the importance of mtROS in bacterial activation of the AIM2 inflammasome; differential activation of AIM2 after Francisella infection was due to the sensitivity of each isolate to ROS. The ROS present in the early stages of Francisella novicida (Fn) infection can activate the AIM2 inflammasome independently of NLRP3 and NADPH oxidase, while mitochondrial ROS (mtROS) is crucial for Fn stimulation of the inflammasome (Crane et al., 2014). The roles of BAX, BAK1, and MOMP in routine inflammasome activation remain to be fully elucidated, with multiple possible mechanisms. Initially, bacterial lipopolysaccharide plus ATP activation of the NLRP3 inflammasome was proposed to be independent of BAX, BAK1, etc., but later it was found that activation of BAX and BAK1 can promote CASP8-dependent NLRP3 inflammasome activation and trigger NLRP3-independent pathways. BAX and BAK1-independent inflammasome activation may involve gasdermin cleavage, and a preprint linked Mitochondrial permeability transition (MPT) to at least some instances of NLRP3 inflammasome signaling (Chauhan et al., 2018; Guarnieri et al., 2023; Platnich et al., 2018; Rodríguez-Gómez et al., 2020; Rogers et al., 2019; Vince et al., 2018; Wang et al., 2017). In summary, mtDNA and mitochondrial ROS, as major injury-related molecular patterns of inflammasomes, have pathways that are intertwined with molecular machines that regulate regulatory cell death, providing potential targets for the treatment of inflammation-related diseases.

Mitochondria represent the principal cellular source of reactive oxygen species (ROS), with mitochondrial ROS (mtROS) serving as essential signaling molecules that regulate macrophage polarization and mediate inflammatory signal transduction pathways (Li et al., 2025b). Upon stimulation with lipopolysaccharide (LPS) or Toll-like receptor (TLR) ligands, macrophages activate mitochondrial reactive oxygen species (mtROS) production via the mitochondrial electron transport chain (ETC). This process is tightly regulated by the TNF receptor-associated factor 6 (TRAF6)–evolutionarily conserved signaling intermediate in Toll pathways (ECSIT) complex, which localizes to the outer mitochondrial membrane and interacts with the mitochondrial citrate carrier (SLC25A1). Additionally, immunoresponsive gene 1 (IRG1) enhances mtROS generation by promoting oxidative phosphorylation within phagosomes. Elevated mtROS levels subsequently drive macrophage polarization toward the proinflammatory M1 phenotype, exerting direct antibacterial effects while serving as critical signaling molecules to stimulate the secretion of proinflammatory cytokines, including TNF-α (Tan et al., 2016).

Under conditions of cellular stress or dysfunction, mitochondrial reactive oxygen species (mtROS) secretion increases, exacerbating mitochondrial impairment. The regulatory effects of mtROS on macrophage polarization are dose-dependent and exhibit functional duality: low levels are indispensable for promoting anti-inflammatory M2 polarization, whereas high concentrations drive proinflammatory M1 polarization (Tian Z. et al., 2025). In rheumatoid arthritis (RA), the hyperactivation of the NF-κB/HIF-1α signaling axis elevates mitochondrial reactive oxygen species (mtROS) levels, thereby exacerbating M1 macrophage polarization and promoting disease progression. Additionally, the ROS-dependent Ataxia telangiectasia mutated (ATM)–Checkpoint kinase 2 (Chk2) pathway has been implicated in driving aberrant M1 polarization in RA (Li et al., 2025b). The role of mitochondrial reactive oxygen species (mtROS) in M2 anti-inflammatory macrophage polarization remains controversial. Some studies demonstrate that under Th2 cytokine or natural compound stimulation, mtROS promote M2 polarization via AMP-activated protein kinase (AMPK) activation and mitophagy induction. Conversely, other evidence suggests that reducing mtROS levels facilitates the phenotypic transition from M1 to M2 macrophages, thereby promoting inflammation resolution.

### 5.3 Other PRR signaling pathways

Besides cGAS and the inflammasome pathway, mtDNA and other mitochondrial components can trigger inflammatory responses via various pattern recognition receptors (PRRs) (West and Shadel, 2017). Naked or protein-bound mtDNA is a potent activator of TLR9 and the receptor for advanced glycation end products (AGER, also known as RAGE), both of which are abundant in the endosomal compartment of myeloid cells. Naked mtDNA, due to its high similarity to bacterial DNA, primarily acts as a TLR9 agonist; mtDNA that forms protein complexes can exert immunostimulatory effects after binding to TLR9 or AGER. Recombinant TFAM can promote cytokine secretion, but its underlying mechanisms and pathophysiological significance require further investigation.

Activation of inflammatory pathways often requires mtDNA to be released into the extracellular microenvironment as a result of regulatory cell death (RCD) to exert its effects, but there are exceptions. Cells such as plasmacytoid dendritic cells can initiate endosomal TLR9 signaling in cases of mild mitochondrial dysfunction; breast cancer cells release mitochondrial-derived vesicles (MDVs) carrying mtDNA due to metabolic alterations, triggering autocrine and/or paracrine TLR9 activation, although the reason why PRKN excludes Mitochondrial damage−associated molecular pattern (mtDAMP) remains unclear. During sepsis, monocytes actively generate MDVs containing mtDAMP, and in hormone-resistant breast cancer models, mtDNA-containing MDVs can be horizontally transferred to tumor cells, indicating the existence of RCD-independent mtDNA release and DAMP-like effects.

### 5.4 RIG-I pathway

Another inflammatory pathway that can be activated by mitochondria involves RIG-I-like receptors (RLRs), which respond to exogenous, altered, or aberrantly located RNA (Hur, 2019). Mitochondria not only provide the RNA source for RLR activation but also support RLR signaling by carrying mitochondrial antiviral signaling proteins on their outer membrane. Studies have shown that after depletion of the polynucleotide phosphorylase PNP enzyme (PNPT1), mitochondria release mtRNAs that can activate RLRs, and mtDNA fragmentation can drive RIG-I activation (Coomans de Brachène et al., 2021). The reasons why specific mtRNAs preferentially activate RLRs require further investigation and may be related to cell type-specific differences. Li et al. found that in the context of viral infection, the mitochondrial protein Era like 12S mitochondrial rRNA chaperone 1 (ERAL1) can translocate from mitochondria to the cytoplasm, promoting MAVS polymerization in a RIG-I and Melanoma differentiation-associated protein 5-dependent (MDA5-dependent) manner through the BAX/BAK pores, thereby actively regulating the antiviral response (Li et al., 2021). MAVS can regulate cell fate and inflammatory responses, consistent with the immunological characteristics of RCD molecular regulation as shown in numerous studies.

### 5.5 SMAC (DIABLO)-mediated NF-κB pathway

Studies have found that other components of mitochondria also promote inflammatory responses. The release of Second mitochondria-derived activator of caspases (SMAC), a caspase activator downstream of MOMP, is transduced into pro-apoptotic and pro-inflammatory signaling pathways via members of the Inhibitor of Apoptosis Protein (IAP) family of apoptosis-inhibiting proteins. Inhibition of IAP by cytoplasmic SMAC (and SMAC mimicry pharmacological agents) stabilizes Mitogen-activated protein kinase kinase kinase 14 (MAP3K14, also known as NIK), thereby shifting NF-κB signaling from a canonical pathway to a non-canonical pathway under the coordination of the BAX-BAK1 oligomer (Gyrd-Hansen and Meier, 2010). Correspondingly, the deletion of the genes Baculoviral IAP repeat-containing protein 2 (Birc2) and Birc3 encoding IAP in adult mice leads to aberrant cell death and inflammation; these phenotypes can be completely eliminated by the pharmacological inhibition of Caspase-8 combined with NIK deletion (Zhang et al., 2019). Researchers have found that cytoplasmic mtDNA or mtRNA typically triggers multifaceted TANK-binding kinase 1 (TBK1)-dependent inflammatory responses, while SMAC-driven inflammation appears to primarily involve altered NF-κB signaling (Marchi et al., 2023).

In summary, numerous mitochondrial components and products can promote inflammation through various mechanisms (**Table 2**). Mitochondria play an important role in regulating inflammatory responses, and eukaryotic cells have evolved multiple mechanisms to regulate mitochondrial-induced inflammatory responses.

**Table 2:** Summary of mitochondrial signaling pathways regulating inflammation.

Molecular mechanism	Main ingredients	Mechanism of action and regulatory process	Related diseases or research models	Targeted drugs	Mechanism of action	References
cGAS-STING1 pathway	cGAS, STING1, mtDNA	cGAS recognizes leaked mtDNA in the cytoplasm, catalyzes the production of cGAMP, activates STING1, and triggers an inflammatory response. It is inhibited by apoptotic caspases, and different forms of dsDNA exhibit varying abilities to activate cGAS.	Metabolic inflammatory diseases, autoimmune diseases	PF-06928125	Inhibiting the cGAS-STING1 pathway reduces the inflammatory response triggered by mtDNA leakage.	(Decout et al., 2021)
Inflammatory bodies	NLRP3, AIM2, mtDNA, ROS	mtDNA and ROS activate the NLRP3 and AIM2 inflammasomes, promoting the release of inflammatory factors. Oxidation is crucial for mtDNA-induced inflammasome signaling.	Atherosclerosis, infectious diseases	MCC950 (CP-456,773),CY-09, C77	Blocking the interaction between NLRP3 and NIMA−related kinase 7 (NEK7) inhibits ASC polymerization and Caspase-1 activation.	(Saxena et al., 2010)
TLR9/RAGE signaling pathway	TLR9, RAGE, mtDNA	Naked or protein-bound mtDNA acts as an agonist of TLR9 and RAGE, triggering inflammatory responses. TLR9 is primarily expressed in the endosome compartment and recognizes mtDNA with similar bacterial DNA structures.	Sepsis and breast cancer models	TTP488 (Azeliragon)	Blocking TLR9/RAGE signaling reduces mtDNA-triggered inflammatory responses.	(Hur, 2019)
RIG-I pathway	RIG-I, MAVS, mtRNA	MAVS on the outer mitochondrial membrane supports RIG-I-like receptor signaling, and mtRNA activates RIG-I. Specific mtRNA types preferentially activate RIG-I, which may be related to cell type specificity.	Antiviral response in the context of viral infection	Parkin (PRKN)	Regulating the RIG-I signaling pathway affects mitophagy and antiviral responses.	(Hur, 2019)
SMAC-mediated NF-κB pathway	SMAC, IAP family, NF-κB	SMAC release downstream of MOMP transduces into pro-apoptotic and pro-inflammatory signaling pathways by inhibiting IAP family members. Stabilization of MAP3K14 (NIK) shifts NF-κB signaling from a classical pathway to a non-classical pathway.	Cell death and inflammation-related diseases	LCL161	Inhibition of the IAP family promotes activation of the non-canonical NF-κB pathway and induces tumor cell apoptosis.	(Gyrd-Hansen and Meier, 2010)

## 6 Challenges and future directions

Currently, exploring the relationship between mitochondria and inflammation regulation, as well as disease treatment, faces numerous complex and pressing challenges. Regarding mechanistic analysis, although substantial evidence suggests that mitochondrial dysfunction-driven inflammatory responses are associated with the pathogenesis of various human diseases, established mechanistic connections are severely lacking, significantly limiting our in-depth understanding of the nature of diseases. Furthermore, the functional interconnections between MOMP and MPT are not fully elucidated, and the major roles and interactions of autophagy and apoptotic caspases in regulating mtDAMP-driven inflammation are poorly understood. Key molecular details, such as the interaction between mtDNA and inflammasomes and the interaction of MAVS in maintaining the mitochondrial outer membrane, remain to be clarified. In addition, many experimental strategies used to analyze the role of mtDAMP in inflammatory responses may obscure results due to the numerous alterations imposed on cells, necessitating the development of more sophisticated experimental tools. In terms of clinical translation, the bioavailability and tissue-specific delivery of mitochondrial-targeted drugs face challenges, and personalized mitochondrial function assessment standards are not yet perfected. These factors constitute significant obstacles to translating research findings into practical therapeutic approaches.

To address these challenges, future research needs to focus on multiple directions. In terms of mechanism analysis, advanced technologies such as single-cell sequencing and spatial metabolomics can be used to delve deeper into mitochondrial-related mechanisms. For example, single-cell sequencing can reveal mitochondrial heterogeneity in macrophage subsets, while spatial metabolomics can pinpoint the microenvironment-dependent nature of mitochondrial stress, providing more precise data support for a comprehensive understanding of the role of mitochondria in inflammation and disease. In terms of clinical translation, efforts are focused on improving the bioavailability and tissue-specific delivery of mitochondrial-targeted drugs, developing novel carriers such as Szeto-Schiller (SS)-31 peptide to achieve mitochondrial-specific enrichment of drugs in specific tissues; and establishing personalized mitochondrial function assessment standards. By combining Seahorse analysis with metabolomics, mitochondrial function can be accurately assessed, providing a basis for personalized treatment. Furthermore, interdisciplinary innovation will bring new breakthroughs to this field. Artificial intelligence can be used to predict mitochondrial stress patterns and disease risk, constructing predictive models that automatically identify high-risk populations; and organoid models can be used to simulate macrophage-mitochondrial interactions in complex tissues, providing a more physiologically relevant experimental platform for studying disease mechanisms and developing treatments, thus propelling mitochondrial-targeted therapy from basic research to clinical application.

Currently, mitochondria-targeted therapeutic strategies are undergoing extensive preclinical evaluation across multiple disease models, including sepsis, neurodegenerative disorders, and diabetic nephropathy, with several candidates advancing into early-phase clinical trials—providing critical evidence for mechanism-based translational research. Mitochondria-targeted antioxidants, engineered to selectively penetrate and accumulate within the mitochondrial phospholipid bilayer, represent a precision-medicine approach by modulating mtROS production at its source, thereby minimizing off-target effects and enhancing therapeutic efficacy. This strategy holds particular promise in sepsis, where dysregulated mtROS contributes to multi-organ dysfunction; key compounds include MitoQ (the most extensively studied), MitoVitE, Visomitin (SkQ1), and SS-31 (Hong et al., 2024). Preclinical studies demonstrate that MitoQ effectively mitigates oxidative stress and improves clinical outcomes in sepsis-induced organ dysfunction. SS-31, a mitochondria-targeted peptide that binds to mitochondrial cardiolipin to suppress mtROS production and stabilize mitochondrial ultrastructure, exhibits significant protective effects in experimental models of sepsis and inflammatory disorders (Chavez et al., 2020). Mechanistic studies indicate that enhancing mitochondrial function or suppressing mtROS—through pathways involving Sirtuin 3 (SIRT3) activation, mesenchymal stem cell (MSC) therapy, or targeted antioxidants—can mitigate pathological responses and organ injury in sepsis. Despite these advances, significant challenges remain. Achieving precise tissue-specific delivery, particularly to the brain, continues to be a major obstacle. Moreover, the long-term effects on mitochondrial dynamics, cellular health, and systemic homeostasis require further investigation. Future research should prioritize (1) optimizing delivery systems for tissue selectivity, (2) evaluating long-term safety and efficacy across heterogeneous patient populations, and (3) exploring synergistic combinatorial strategies to enhance therapeutic robustness. These efforts are critical for translating mitochondria-targeted interventions into clinically viable treatments for sepsis and other mtROS-associated diseases (Silwal et al., 2020; Wang et al., 2025).
